# The Complement Cascade and Renal Disease

**DOI:** 10.1007/s00005-013-0254-x

**Published:** 2013-09-13

**Authors:** Katarzyna Kościelska-Kasprzak, Dorota Bartoszek, Marta Myszka, Marcelina Żabińska, Marian Klinger

**Affiliations:** Department of Nephrology and Transplantation Medicine, Wroclaw Medical University, Wrocław, Poland

**Keywords:** Complement cascade, Renal disease, Kidney injury

## Abstract

Serum complement cascade, a part of innate immunity required for host protection against invading pathogens, is also a mediator of various forms of disease and injury. It is activated by classical, lectin, and alternative pathways that lead to activation of C3 component by C3 convertases, release of C3b opsonin, C5 conversion and eventually membrane attack complex formation. The tightly regulated activation process yields also C3a and C5a anaphylatoxins, which target a broad spectrum of immune and non-immune cells. The review discusses the involvement of the complement cascade in kidney disease pathogenesis and injury. The role of the complement pathways in autoantibody-mediated forms of glomerulonephritis (lupus nephritis, anti-glomerular basement membrane disease, anti-neutrophil cytoplasmic autoantibody-induced or membranoproliferative glomerulonephritis, membranous nephropathy), C3 glomerulopathy, atypical forms of hemolytic uremic syndrome, ischemic-reperfusion injury of transplanted kidney, and antibody-mediated renal allograft rejection are discussed. The disturbances in complement activation and regulation with underlying genetics are presented and related to observed pathology. Also promising strategies targeting the complement system in complement-related disorders are mentioned.

## Introduction

Complement cascade is a set of soluble and cell-bound components, regulators, and receptors, which can not only be activated in response to pathogens, but also due to the presence of autoantibodies, apoptotic, necrotic or ischemic cells, and tissues. It has evolved to protect against infections through promoting phagocytosis of complement opsonized pathogens, immune cell chemotaxis, and direct pathogen lysis, but its activity, normal or disturbed, plays also a significant role in pathogenesis of a number of non-infectious diseases. The aim of the current review is to discuss the role of complement activation in pathogenesis of kidney disease and injury.

The complement system integrates the interactions of leukocytes, platelets, and tissues in inflammatory response and can be activated via the classical, lectin or alternative pathways (Sarma and Ward 2011). The activation trigger is detection of threats, such as pathogens, altered self molecules or surfaces (as a result of injury, hypoxia, virus infection or malignancy), and apoptotic cells. The key molecules of initiation of the cascade are:

- C1q, which binds immunoglobulin complexes (as part of C1 complex) or surface of the pathogen, and initiates the classical pathway;
- Mannose-binding lectin (MBL) involved in recognition of mannans on the surface of various microorganisms in the lectin pathway;
- And properdin, a positive regulator of the alternative pathway.

**Fig. 1:**
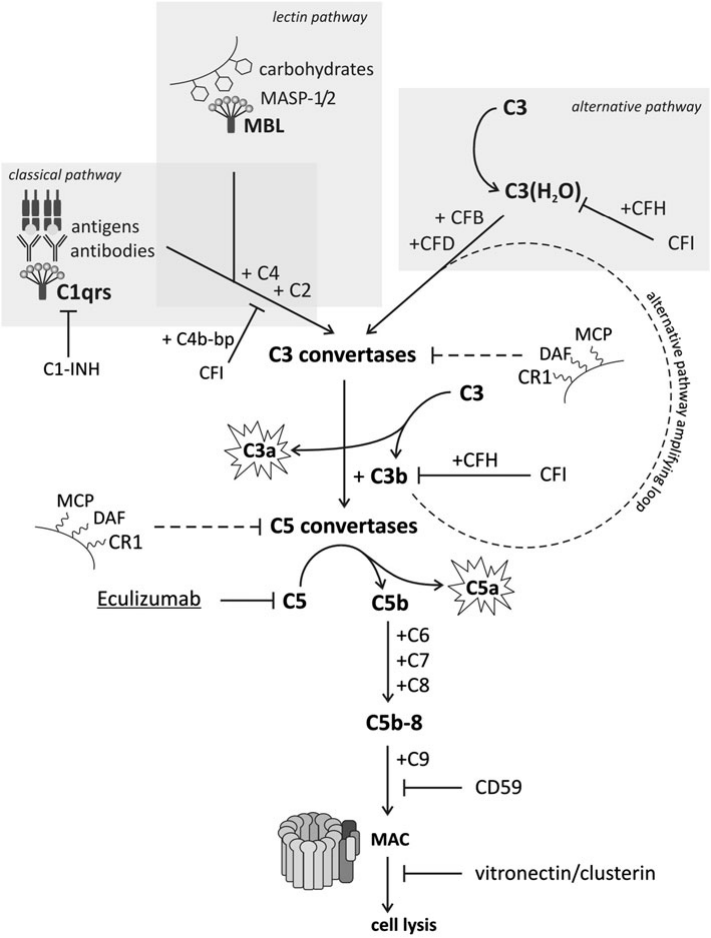
Complement activation pathways. Classical pathway is activated by C1 assembled on antigen-bound antibodies or in an antibody-independent manner by some viruses or Gram-negative bacteria, and the lectin pathway by mannose-binding lectin (MBL) attached to carbohydrates on the surface of microorganisms. Both pathways converge to release the common C3 convertase (C4bC2b). The alternative pathway is constantly activated at a tightly controlled level and generates C3 convertase (C3(H_2_O)Bb/C3bBb) that release C3b at low level, which can readily initiate amplification. The assembled convertases cleave C3 to anaphylactic and antimicrobial C3a and C3b, an opsonin that is deposited on nearby surfaces and serves to amplify the activation signals. C3b also serves to generate the next generation C5 convertases, which further activate the cascade, leading to terminal membrane attack complex and target cell lysis

All the three pathways lead to activation of C3 component by C3 convertases, release of C3b opsonin, C5 conversion, and eventually membrane attack complex (C5b-9) formation (Fig. 1). C5b-9 disrupts the phospholipid bilayer of target cells causing cell injury and necrosis. The complex is also capable of activating neutrophils, endothelium, and epithelium.

The complement activation process yields also C3a and C5a anaphylatoxins, potent inflammatory mediators, which target a broad spectrum of immune and non-immune cells (Klos et al. 2009). C5a is a powerful chemoattractant for macrophages, neutrophils, activated B and T cells, basophils and mast cells. Anaphylatoxins regulate vasodilation, increase the permeability of small blood vessels, induce contraction of smooth muscles, oxidative burst in macrophages, neutrophils and eosinophils, release of histamine by basophils and mast cells. They also regulate tissue regeneration and fibrosis.

The alternative pathway is continuously activated at a basal level due to the thioester bond of C3 slowly hydrolyzing and converting inactive native C3 to a functionally active C3b-like molecule, C3(H_2_O). C3(H_2_O) serves as a subunit of the initial C3 convertase, and its continuous production is a basis of the “tick-over” phenomenon of the alternative pathway (Bexborn et al. 2008). C3b, the product of the reaction catalyzed by the C3 convertase, is further involved in the formation of C3 convertase complex, making a positive feedback loop of the alternative pathway.

The complement activation is strictly regulated to prevent autoaggressive damage of the host cells both in a fluid and solid phase. A number of soluble regulators are involved in the control of the complement activation. C1-inhibitor (C1-INH) prevents the autoactivation of the initial complex of classical pathway. C4b-binding protein is a decay-accelerating factor for the classical pathway C3 convertase and a cofactor for factor I (CFI) cleavage of C4b opsonin. The similar activity in the alternative pathway is provided by factor H (CFH), which is involved in decay of convertase and C3b inactivation by CFI. Clusterin and vitronectin act on terminal complexes preventing their insertion into the cell membranes. Also carboxypeptidase *N* is a part of fluid-phase regulatory activity of the three pathways, acting as anaphylatoxin inhibitor. Surface-bound regulatory proteins, CR1 (C3b receptor), membrane cofactor protein (MCP, CD46), and decay-accelerating factor (DAF, CD55) function to shorten the half-life of cell surface assembled C3 and C5 convertases. Complement-mediated injury will proceed if the triggered activation outweighs the inhibitory potential of the pathway regulators.

In the setting of kidney disease pathogenesis focused in this review, the complement cascade is involved in autoantibody-mediated forms of glomerulonephritis, C3 glomerulopathy, atypical forms of hemolytic uremic syndrome, ischemic-reperfusion injury of transplanted kidney, and antibody-mediated renal allograft rejection. Different sites of defective complement regulation or deficiency of particular components lead to various manifestations of complement-related disease and influence its outcome. The major source of serum complement is liver, however, it is known that other parenchymal tissues can also release and activate complement under certain circumstances. Most of the alternative and classic pathway components, needed for complement activation, are expressed in renal tissue (Song et al. 1998). Local renal production of complement serves as a signal for kidney inflammation and repair and is observed due to numerous homeostatic and pathological factors with ischemia–reperfusion injury as an example (Sacks and Zhou 2008).

## Glomerulonephritis

Glomerulonephritis is one of the most common causes of chronic kidney disease and end-stage renal failure in the world. It is not related to a single syndrome, but rather describes the general phenotype, characterized by glomerular inflammation and cell proliferation, leading to a number of clinical consequences, such as hematuria, proteinuria, and reduced glomerular filtration rate.

The presence of autoantibodies and the autoantibody-mediated involvement of classical pathway of the complement cascade is the cause of glomerulonephritis related to systemic diseases, such as lupus, anti-glomerular basement membrane (anti-GBM) disease, anti-neutrophil cytoplasmic autoantibody (ANCA)-associated vasculitides, Henoch-Schönlein purpura or as kidney restricted membranous nephropathy (MN), membranoproliferative glomerulonephritis (MPGN), and IgA nephropathy. On the other hand, the pathophysiological background of C3 glomerulopathy is the uncontrolled systemic activation of the alternative pathway of the complement cascade.

## Systemic Lupus Erythematosus

Systemic lupus erythematosus (SLE) is a systemic autoimmune disease characterized by immune response against nuclear antigens and the presence of circulating immune complexes (Tsokos 2011). Much of pathophysiology of SLE is related to immune complexes and their deposition in affected organs, as in glomeruli in the case of kidney involvement. However, there is a range of immunological abnormalities in SLE, such as disturbances in the activation of T, B and dendritic cells, the subsequent production of autoantibodies, and the above mentioned formation and deposition of immune complexes causing multi-organ inflammatory injury. Lupus nephritis, developing due to immune complex deposition in glomeruli, is one of the most threatening manifestations of SLE and a major predictor of poor prognosis. Complement is a major player in removal of pathological immune complexes, but on the other hand, its activation products promote inflammation, fibrosis, and tissue injury, particularly if activation is prolonged.

In vitro and in vivo studies demonstrated that patients with SLE present an impaired clearance of apoptotic cells. These abnormalities lead to constant immune system exposure to autoantigens and subsequent development of autoimmunity, mostly directed against nuclear antigens (Bijl et al. 2001). Normally, the autoreactive B cells are eliminated after complement opsonized autoantigens are bound to CR1 and CR2. Deficiency in complement components lead to circulating autoreactive B cells and sustained autoreactive antibody production (Truedsson et al. 2007). The impairment of removal of immune complexes formed between autoantibodies and self-antigens is considered a key mechanism underlying the development of systemic lupus erythematosus. During disease flare, an increased consumption of C1q and C4 complement proteins is associated with a reduced density of complement receptor CR1 (CD35) on the erythrocyte surface. Binding to CR1 receptor is a key step in removal of immune complexes from the circulation. Downregulated CR1 expression in lupus leads to elevated levels of circulating immune complexes and their potential deposition in tissues (Iida et al. 1982).

Because clearance of immune complexes depends largely on early complement component C1q, homozygous C1q deficiency is a strong genetic risk factor in SLE, although, it is a rather rare background for SLE development overall. Hereditary C1q deficiency is caused either by a lack of synthesis of C1q (about 60 % of cases) or through the synthesis of low molecular weight nonfunctional protein, LMWC1q. The highest incidence and most severe symptoms are associated also with other C1 complex deficiencies or total absence of C4. Approximately 75–90 % of patients with homozygous deficiency of C1 or C4 suffer from SLE or SLE-like disease (Taylor et al. 2000). The anti-C1q autoantibodies are reported in 30 % of patients with unselected SLE and show a strong inverse correlation with levels of C3 and C4. They are strongly associated with renal involvement in SLE and related to severe course of the disease (Marks and Tullus 2011). Increasing levels of anti-C1q antibody titers have been suggested predictive for the exacerbation of the disease (Seelen et al. 2003a). However, they are not considered pathogenic by themselves as their influence is seen only in the presence of complement-fixing autoantibodies (Trouw et al. 2004).

The initiation of classical pathway is regulated by the interaction of C1-INH with the C1 complex. In SLE patients, autoantibodies directed against C1-INH are observed, however, their presence is not linked to renal involvement in the disease course (Meszaros et al. 2010).

Mannose-binding lectin (MBL) is structurally and functionally similar to C1q, and this fact led to the hypothesis that MBL-deficient individuals may also be predisposed to SLE development. Low MBL concentration occurs as a result of polymorphisms in the MBL gene, increased MBL consumption during disease, or due to the presence of antibodies directed against MBL. The influence of MBL genetics on SLE susceptibility has been suggested (Glesse et al. 2011; Lee et al. 2005; Sandrin-Garcia et al. 2011). The presence of anti-MBL IgG autoantibodies was also observed and their serum level correlated with serum MBL level (Mok et al. 2004). However, not all the studies confirmed the relation of anti-MBL antibodies to lupus development and activity (Mok et al. 2004; Pradhan et al. 2013; Shoenfeld et al. 2007). Contrary to anti-C1q antibodies, anti-MBL antibodies have not been proved to influence lupus nephritis development (Seelen et al. 2003b).

## Membranoproliferative Glomerulonephritis and C3 Glomerulopathy

Membranoproliferative glomerulonephritis (MPGN) is an uncommon cause of chronic nephritis, characterized by a pattern of glomerular injury involving proliferation of mesangial and endothelial cells, expansion of mesangial matrix, thickening of peripheral capillary walls, and mesangial interposition into the capillary wall (Smith and Alpers 2005).

Formerly, the MPGN was classified on the basis of immunopathology and ultrastructure analysis of the kidney, in particular glomerulus, into three subtypes: MPGN type I, II (dense deposit disease) and III. Recently, the classification of MPGN has been greatly reviewed and more strictly focused on the type of immune activation underlying the disease (D’Agati and Bomback 2012; Sethi et al. 2012). The newly emerged C3 glomerulopathy designation describes the MPGN type microscopic image with isolated C3 deposits, encompassing dense deposit disease and some formerly classified MPGN I and III cases with C3 deposits without clear staining for immune complexes. With this reclassification, the MPGN types I and III are those caused by the presence of subendothelial, mesangial, or also subepithelial, and intramembranous immune complexes with C1q and are associated with the classical pathway of complement activation. On the contrary, the pathophysiologic basis of C3 glomerulopathy is associated with the uncontrolled systemic activation of the alternative pathway of the complement cascade. In most patients, loss of complement regulation is caused by C3 nephritic factor (C3NeF), an IgG autoantibody directed against the neo-epitope of C3 convertase of the alternative pathway. C3Nef binds assembled C3 convertase making it resistant against inactivation by factor H and leads to an uncontrolled complement activation through the alternative pathway, resulting in an acquired deficiency of C3. Also non-C3NeF C3 convertase stabilizing autoantibodies have been reported that were directed against C3b and factor B (CFB) (Chen et al. 2011). On the regulatory level, mutations in the CFH gene and anti-CFH autoantibodies have been identified in the C3 glomerulopathy patients and related to pathogenesis of the disease (Appel et al. 2005; Goodship et al. 2012; Licht and Fremeaux-Bacchi 2009; Zhang et al. 2012). CFH mutations related to C3 glomerulopathy are particularly those leading to the absence of the functional component in plasma, and as a result to unrestricted alternative pathway activation (Zipfel and Skerka 2009).

## ANCA-Associated Vasculitides

The ANCA-associated vasculitides (AAVs) include Wegener’s granulomatosis (WG), microscopic polyangiitis (MPA) and its renal limited subset idiopathic necrotizing crescentic glomerulonephritis (NCGN), or Churg–Strauss syndrome (CSS). These diseases are characterized by necrotizing inflammation of small vessels in conjunction with the presence of ANCA directed either to proteinase 3 (PR3) or myeloperoxidase (MPO). The kidneys are the organs, which AAV affect the most. Chen et al. (2009) examined 112 biopsies from patients suffering from pauci-immune ANCA-associated glomerulonephritis. In one third of those biopsies, C3c deposits were detected in glomeruli. Patients with C3c deposition had higher levels of proteinuria and poorer renal function, if compared with patients without C3c deposition. Further research had also shown MAC, C3d and CFB in biopsies from patients with MPO-ANCA-associated pauci-immune necrotizing crescentic glomerulonephritis. This suggests an involvement of alternative pathway of complement activation in AAV (Chen et al. 2009). Also, the elevated plasma and urinary C5a levels indicated complement activation in human AAV, also varying between active and remission phases of the disease (Yuan et al. 2012).

Schreiber et al. (2009) demonstrated that C5a specific receptor, C5aR, expressed on neutrophils is essential in the pathogenesis of ANCA-induced NCGN. Their study showed that generation of C5a in normal human serum might be induced by supernatants from ANCA-stimulated neutrophils. C5a binding to the C5a receptor (C5aR) enhances the influx of neutrophils and their activation, leading to ROS generation and severe necrotizing of vascular wall (Schreiber et al. 2009). The orally administered C5aR antagonist, CCX168, has proved promising in the treatment of ANCA glomerulonephritis in mice. Clinical study on CCX168 therapy in patients with ANCA-associated renal vasculitis is currently in progress (NCT 01363388).

## Membranous Nephropathy

Until recently, the pathogenic mechanisms of MN were based on Heymann’s experimental rat model of nephritis. The model suggested that antibodies targeting podocyte antigens, accumulating as immune deposits, and activation of the complement lead to sublethal injury of the podocyte, as well as proteinuria (Beck and Salant 2010). According to the latest studies, the main causes of MN are circulating antibodies against the M-type phospholipase A2 receptor (PLA2R), a trans-membrane protein located on podocytes. Immunoglobulin G4 (IgG4), the predominant anti-PLA2R IgG subclass, is incapable of activating the classical complement pathway. This suggests that perhaps the alternative or MBL pathway may be involved in the pathogenesis of MN (Beck and Salant 2010). There are reports showing glomerular MBL and C4b deposition in patients with idiopathic MN, which may confirm that disturbances in MBL complement activation pathway are related to pathogenesis of MN (Lhotta et al. 1999; Val-Bernal et al. 2011). Despite recent advances in the diagnosis and treatment of idiopathic MN, pathogenetic mechanism and triggers for anti-PLA2R production are still elusive.

## Goodpasture’s Syndrome

Goodpasture’s syndrome (anti-GBM disease) is rare but serious disease caused by IgG autoantibodies, directed against the GBM. A typical manifestation of anti-GBM disease is rapidly progressive glomerulonephropathy accompanied by pulmonary hemorrhage. Anti-GBM glomerulonephropathy is characterized by deposition of immune complexes along the GBM. These immune complexes contain autoantibodies against GBM proteins, such as collagen type IV and neutral endopeptidase (Foster 2008; Hudson et al. 2003). Binding of anti-GBM autoantibodies to the GBM leads to autoimmune damage characterized by strong activation of the complement (evidenced by deposits of C3), infiltration of leukocytes into the inflamed tissue, and proteinuria. All this leads to the deterioration and loss of renal function. The research shows that the complement system plays a role in renal injury due to Goodpasture’s syndrome by the proinflammatory effect of classical pathway activated C5a and/or cell lysis effect of C5b-9 (Foster 2008; Ma et al. 2013; Turnberg and Cook 2005).

## Henoch-Schönlein Purpura Nephritis and IgA Nephropathy 

IgA nephropathy and Henoch-Schönlein purpura nephritis (HSPN) are common glomerular disorders that can potentially progress to end-stage renal disease. Both of the two diseases are characterized by IgA deposition within the glomeruli, and the IgA concentrations in serum increase during the acute phase of the disease. IgA deposits in HSPN and IgA nephropathy are frequently associated with complement factors––most often C3, properdin, and MAC. It has been shown that alternative and lectin pathways of complement activation participate in pathogenesis of those diseases (Chen et al. 2010). Hisano et al. (2001, 2005) showed that in patients with IgA nephropathy mesangial deposits of C3c, C4, MBL and MASP-1 accompanied mesangial IgA1 and IgA2 deposits. As there were no C1q deposits, the complement must have been activated by an alternative and lectin, but not classical pathway. In other patients, only mesangial deposits of IgA and C3c were present, without C4, MBL and MASP-1, which suggest that in some cases, activation takes place only via the alternative pathway (Hisano et al. 2001, 2005). Similar results were obtained in patients with IgA nephropathy. Patients with positive staining for mesangial C4d, indicating that complement is activated through the lectin pathway, had worse renal function and demanded more aggressive treatment (Espinosa et al. 2009). Recent study by Gharavi et al. (2011) showed that the presence of CFHR1 and CFHR3 deletion genotype variant may have a protective effect in IgA nephropathy. It might be due to competitive action of CFH and CFHR1 proteins, so that the loss of CFHR1 increases the strength of CFH regulatory impact, leading to reduction of inflammation.

## Atypical Hemolytic Uremic Syndrome

The hemolytic uremic syndrome (HUS) is a heterogeneous disease of the microvasculature that is characterized by the triad of microangiopathic hemolytic anemia, thrombocytopenia and acute renal failure. Most often typical HUS is triggered by verocytotoxin-producing bacterium, such as *Escherichia coli* (O157:H7, O111:H8, O103:H2, O123, O26) or *Shigella dysenteriae* and manifests with diarrhea, which is often bloody. The incidence of typical HUS is mainly observed in children under 3 years of age and usually is associated with good long-term prognosis. However, ~10 % of the hemolytic uremic syndrome is the familial or sporadic atypical form (aHUS) without any evidence of bacterial infection. The latter may be associated with pregnancy, malignancies, transplantation, viral agents or drugs (e.g. immunosuppressive, antiplatelet, antineoplastic) (Besbas et al. 2006; Loirat and Fremeaux-Bacchi 2011; Noris and Remuzzi 2009). In contrast to the typical form of HUS, aHUS can occur at any age. In case of aHUS, the prognosis is poor with mortality rate of about 25 %. More than half of the cases have relapses and progress to end-stage renal disease. The central role in the pathogenesis of aHUS is played by defects in regulation of the alternative complement pathway. In over 60 % of cases, mutations have been identified in genes encoding complement regulatory proteins: CFH, CFI, MCP, thrombomodulin (THBD), and complement activators: CFB and C3. An uncontrolled activation of complement causes a damage of endothelium, thrombosis and microangiopathic hemolytic anemia (Loirat et al. 2008; Roumenina et al. 2011).

CFH is the most important serum glycoprotein in regulation of the alternative pathway, and is a cofactor for CFI, the C3b-inactivating enzyme. CFH mutations are observed in 25–30 % patients with aHUS (Kavanagh and Goodship 2011). CFH consists of 20 homologous short consensus repeats (SCRs) and two C3b binding sites. Genes encoding CFH are localized on chromosome 1 at 1q32. The most frequent mutations accumulate in C-terminal region in SCR 19 and 20. Up to now, more than 80 mutations have been defined. Only 15–20 % of CFH mutations were homozygous and usually correlated with low C3 and CFH plasma concentrations (Kavanagh et al. 2008; Loirat and Fremeaux-Bacchi 2011; Moore et al. 2010). In addition, anti-factor H antibodies have been reported in 6–10 % of patients with aHUS. These antibodies bind to SCR 19 and 20 and reduce CFH binding to C3b and cell surfaces (Skerka et al. 2009).

MCP (CD46) also serves as a cofactor for factor I. It is expressed on the surface of various cells and cleaves C3b and C4b. Mutations in MCP have been identified in ~15 % of patients with aHUS and most frequently they are heterozygous. Reduction in MCP expression is reported in over 80 % of cases with mutations in this gene (Loirat et al. 2008; Provaznikova et al. 2012; Roumenina et al. 2011).

Genetic disorders are rarely (5–10 % of aHUS patients) related to serine protease factor I, which regulates three complement pathways. All observed mutations were heterozygous and disrupted cofactor activity or secretion of protein. In approximately 20–30 % of patients, both C3 and CFI concentrations were decreased (Bienaime et al. 2009).

Relatively rare gain-of-function mutations in genes encoding the complement activators C3 and factor B, have been demonstrated in 2–10 and 1–4 % of patients, respectively. About 3–5 % of patients with aHUS carry heterozygous THBD mutations (Delvaeye et al. 2009).

It is recognized that up to 12 % of patients have mutations in more than one gene, usually in CFH with either MCP or CFI (Maga et al. 2010). Genetic disorders associated with aHUS are predisposing rather than directly causal. It is estimated that about 50 % of carriers of CFH, CFI and MCP mutations develop the disease. The course and outcome of aHUS is dependent on the kind of mutations. The worst prognosis is related to CFH, CFI and C3 mutations. In 60–70 % of cases, the loss of renal function, also ESRD, or death during the acute phase is reported. Conversely, the risk of developing ESRD is low in patients with MCP mutations with complete remission observed in 80–90 % of cases (Kavanagh and Goodship 2010; Noris and Remuzzi 2009).

The progress in the understanding of the molecular mechanisms underlying the pathogenesis of aHUS has opened the new way to investigation and treatment. Currently, plasma therapy (plasma exchange/plasma infusion) is the recommended first-line therapy for aHUS. Recent trails confirm efficacy of eculizumab, a humanized monoclonal antibody directed against the complement protein C5. Nurnberger et al. (2009) presented a case of patient with CFH mutation and aHUS relapse after second renal transplantation that was resistant to plasmapheresis treatment. The use of eculizumab resulted in inhibition of hemolysis, increased platelet counts, and improvement of renal function. Nester et al. (2011) proposed the use of eculizumab and plasmapheresis pre-emptively as a part of renal transplant protocol for the treatment of aHUS. Eculizumab has also been used for the treatment of typical shiga-toxin induced HUS. Lapeyraque et al. (2011) published cases of three pediatric patients with severe HUS that did not respond to dialysis and plasma exchange but improved significantly after administration of eculizumab. This report prompted the use of eculizumab in HUS cases during the outbreak in Germany in 2011 (Kielstein et al. 2012). The recombinant CFH and complement blockers, which inhibit the complement activation at the endothelium surface, are also under investigation as potential therapeutic agents (Loirat and Fremeaux-Bacchi 2011).

The complement pathway dysregulation as an underlying mechanism for aHUS development has been largely accepted, however, recently, the deficiency in diacylglycerol kinase ε, DGKE, involved in cell signaling, was related to relapses of acute aHUS in subjects treated with eculizumab-based therapy (Lemaire et al. 2013).

Patients with aHUS who developed ESRD are candidates for renal transplantation. Unfortunately, both the recurrence rate of aHUS after renal transplantation is high (50 %) and graft loss is frequent (80–90 %) (Hirt-Minkowski et al. 2010). The risk of post-transplant recurrence of aHUS depends on a type of complement abnormality (Loirat and Fremeaux-Bacchi 2011; Malina et al. 2012). The highest risk of graft failure is observed in patients with CFH mutations (70–95 %), CFI mutations (45–80 %), and C3 mutations (40–70 %). Contrarily, MCP mutations are associated with a low rate of recurrence (<20 %). In patients with anti-CFH antibodies, the risk of recurrence probably depends on the antibody titer. In the past, combined liver–kidney transplantation was considered due to liver production of complement components (Loirat et al. 2012; Waters and Licht 2011), however, this approach lost importance in eculizumab era.

## Preeclampsia

Pregnancy is associated with a number of immunological changes, which switch between pro- and anti-inflammatory overall immune status to adapt to the presence of fetus, tolerate parental antigens, and induce delivery (Denny et al. 2012). Preeclampsia is a multisystem, heterogeneous disorder that complicates 5–8 % of all pregnancies and is a major cause of maternal and neonatal morbidity and mortality (Denny et al. 2012; Monte 2011; Pennington et al. 2012). Preeclampsia is clinically characterized by vascular endothelial dysfunction, onset of hypertension, and significant proteinuria (protein excretion of ≥300 mg/24 h) developing after 20 weeks of gestation in previously normotensive women. The syndrome can begin earlier in pregnancy with abnormal placental development. The secondary complications of preeclampsia are HELLP syndrome (hemolysis, elevated liver enzymes, low platelets) and eclampsia. Despite extensive research, the etiology and pathogenesis are still not entirely clear. Many studies have confirmed that inflammation and abnormalities in coagulation and angiogenesis are involved in the pathogenesis of preeclampsia. However, SLE or other autoimmune disease patients are more prone to develop this complication (Duckitt and Harrington 2005). Recent reports suggest an importance of complement system activation (Boij et al. 2012; Buurma et al. 2012; Derzsy et al. 2010; Qing et al. 2011; Salmon et al. 2011).

In both normal and pathological pregnancy, complement activation due to a number of stimuli is observed. In a successful pregnancy, the on-going complement activation is strictly regulated and inhibited by membrane regulators: DAF, MCP and CD59. Highly activated complement response that escapes the regulatory potential can lead to pregnancy complications, such as preeclampsia or recurrent miscarriage. Healthy pregnant women had significantly higher C4d, C3a, sC5b-9, C3, C9, factor H levels, and higher C1-INH concentration as compared to non-pregnant women (Derzsy et al. 2010). The levels of C4d, C3a, C5a, C5b-9 observed for preeclampsia women were increased when compared to healthy pregnant ones, and accompanied with lowered C3 level (Boij et al. 2012; Derzsy et al. 2010).

Complement activation products are deposited in placenta, which are assumed to prevent uteroplacental infections (Tedesco et al. 1990). However, deposition of C4d is more pronounced in preeclamptic placentas, which are also characterized by increased expression of regulatory DAF and CD59, probably being a manifestation of fetal feedback trying to counterbalance activation (Buurma et al. 2012). Circulating immune complexes are present in normal pregnancy, and their amount is increased in pathological cases due to the imbalance between formation and removal. The complement can be activated by apoptotic trophoblast cells, which are present in normal pregnancy but increased in an ischemic and oxidatively stressed preeclamptic placenta (Derzsy et al. 2010).

Involvement of alternative pathway complement activation with increased Bb level in early pregnancy period is related to higher preeclampsia risk (Lynch et al. 2008). Also, mutations in complement regulatory proteins MCP and CFI can be found in preeclampsia patients, further supporting the role of complement dysregulation in this pathological state (Salmon et al. 2011).

## Kidney Transplantation

Transplanted kidneys suffer from ischemia–reperfusion (I/R) injury, humoral and cellular rejection, also accompanied by post-transplant infections. The short-term consequences of I/R injury, hyperacute rejection, and infections are mainly governed by innate immunity, and the alloimmunity-mediated by cells or antibodies is due to both innate and adaptive responses. This section will discuss the role the complement system activation has in transplant setting through tailoring the tissue inflammation and recipient alloimmunity.

Transplantation-related vascular and parenchymal cell injury is caused by I/R. The activation occurs mainly due to the alternative pathway resulting from local release of C3 component (Pratt et al. 2000). Some data also suggest the involvement of MBL and pattern recognition of endothelial cell surface ligands exposed due to the ischemia, acting via standard MBL pathway or directly influencing the alternative one without the need for C4 activation (Moller-Kristensen et al. 2005).

The long-term renal allograft survival is largely dependent on the quality of donated organ. The majority of transplanted kidneys are retrieved from cadaveric donors. In fact, these grafts show worse function and lower graft survival after transplantation than those obtained from living donors (Pratschke et al. 2000, 2001). It seems that complement activation might also be involved in the initial injury of deceased allografts. Damman et al. (2010, 2011) found higher gene expression of C3 and increased deposition of C3d in kidney biopsies obtained from deceased grafts. A soluble form of CR1 is under investigation as a potential deceased kidney protecting agent for prolongation of transplant life (Sacks et al. 2013).

Pratt et al. (2002) showed that C3 produced by a graft and by recruited immune cells is a trigger that not only induces early post-reperfusion, but also late rejection associated allograft injury. There are several hypotheses on a role of C3 in allograft rejection. C3 split products, C3b and C3d, deposited on APCs, can increase antigen uptake and its presentation to the T cells, which aids generation of alloreactive clones (Takada et al. 1997). C3-positive APCs (dendritic cells, macrophages and epithelial cells) are shown to potentiate the T cell response in vitro (Pratt et al. 2002). Macrophages deficient for the C3 have impaired capability to stimulate T cells (Li et al. 2004; Peng et al. 2006; Zhou et al. 2006). Also, C3a and C5a binding to T cell receptors may directly stimulate their alloreactivity. The T cell activation by the complement enhances effector T cell expansion by limiting antigen-induced apoptosis (Lalli et al. 2008; Nataf et al. 1999; Schraufstatter et al. 2002). Moreover, recent results have shown that signaling through C5a receptor and C3a receptor could facilitate iTreg-mediated tolerance to alloantigens in humans (van der Touw et al. 2013).

The antibody-mediated renal allograft rejection involves donor-specific antibodies and classical pathway of complement system activation. During its activation, C4 is cleaved and forms the bond with the cell membrane close to the site of antibody attachment. The resultant C4b is quickly degraded by proteolytic enzymes, leaving C4d strongly attached to the cell membrane (Zhou and Sacks 2006). The deposition of C4d in renal grafts was first described in 1993 by Feucht et al. (1993). According to updated Banff’09 criteria for antibody-mediated rejection, C4d diffuse linear deposition in peritubular capillaries (PTC-C4d) with accompanying histological changes is considered as a marker of acute humoral rejection (Sis et al. 2010). There is still a need to look for new methods diagnosing renal rejection, which are less invasive than graft biopsy. Haidar et al. (2012) discovered that the deposition of covalently linked C4d on erythrocytes was more strongly related to histological rejection signs than PTC-C4d staining, but this observation still needs further investigation.

## Future Perspective

Strategies targeting the complement system might become a promising approach in reducing complement-related disorders. The complement system can be targeted at various levels during the cascade. Therapeutic aiming to the complement can act through blockers of circulating complement components or directly on membranes preventing the injury in extra capillary space. It should also be considered whether to target early or late part of a complement cascade. It seems that inhibition at the level of C3 blocks immuno-stimulatory behavior of a complement factors, whereas terminal cascade inhibition would prevent from membrane injury (Zhou and Sacks 2006). The first registered anti-complement drug is eculizumab, anti-C5 humanized antibody, which has been originally approved for the treatment of paroxysmal nocturnal hemoglobinuria and recently also for atypical HUS (Parker et al. 2007). In the nephrology setting, there is a number of successful applications of eculizumab in the treatment of aHUS (Nester et al. 2011; Nurnberger et al. 2009; Zimmerhackl et al. 2010), C3 glomerulopathy (Bomback et al. 2012; Herlitz et al. 2012), antibody-mediated rejection (Gonzalez-Roncero et al. 2012; Stegall et al. 2011; Stewart et al. 2012), and preeclampsia (Burwick and Feinberg 2013). Currently, there are on-going clinical trials to validate the usefulness of eculizumab therapy in ANCA vasculitis and kidney transplantation (http://clinicaltrials.gov). Other anti-complement drugs, which are on their way to renal applications, are compstatin (DeAngelis et al. 2012), recombinant human C1-INH (Tillou et al. 2010), CCX168 (clinical trial NCT 01363388) and soluble form of CR1 (Sacks et al. 2013).
